# ﻿Large Language Models can extract morphological data from taxonomic descriptions, but their stochastic nature makes automation challenging: a test on Australian Asteraceae

**DOI:** 10.3897/phytokeys.261.158396

**Published:** 2025-08-19

**Authors:** Alexander N. Schmidt-Lebuhn, Nunzio Knerr

**Affiliations:** 1 CSIRO, Centre for Australian National Biodiversity Research (a joint venture of Parks Australia and CSIRO), Clunies Ross Street, Canberra ACT 2601, Australia Centre for Australian National Biodiversity Research Canberra Australia

**Keywords:** Asteraceae, Australia, descriptions, Large Language Model, morphology, taxonomy, text mining

## Abstract

Morphological data are critical for taxonomy, evolutionary biology, ecology, and species identification. However, no widely used central database for morphological data exists as it does for DNA sequences or specimen data. Most of these data are “locked up” in taxonomic literature. Various scripted and Natural Language Processing approaches have been explored to automate the extraction of morphological data from taxonomic descriptions. Here, we explore the feasibility of using Large Language Models (LLMs) and Optical Character Recognition (OCR) to rapidly extract data for 51 morphological characters of Australian native and introduced Asteraceae (daisy family) to populate a taxon × character table. ChatGPT 4o was used to process all 1,121 descriptions, which, following currently accepted taxonomy and after accounting for taxa with descriptions in multiple sources, comprise data for 95 genera and 838 species or infraspecific taxa, totalling 945 taxa. The missing data rate is 51.1%. Visual checking of 109 profiles revealed an error rate of 5.8%, a majority of them misapplication of data to the wrong trait based on confusion between different kinds of bracts and between individual involucral bracts and the involucre as a whole. Error rates were lowest for cypsela and pappus characters, at 2.1%. When repeating 109 inferences with the same LLM, 78.9% of the table cells for which at least one replicate had data showed no substantive difference; the main source of inconsistency was 16.7% of those cells having data in only one replicate. When repeating 109 inferences with an open source LLM run on a local computer, results were considerably less reproducible and showed numerous unit errors, irrelevant information being retrieved, and characters being skipped. Our results suggest that while mining of morphological descriptions with LLMs is possible in principle, instructions for the LLM have to be extremely precise. Even then, in contrast to scripting approaches, LLMs are inherently probabilistic. This makes their responses not fully reproducible and their integration into automated workflows difficult. Future work could explore if results can be improved using approaches such as Retrieval Augmented Generation or fine tuning of models on explanations of morphological terminology. The scripts used in the study and the extracted morphological data for Australian Asteraceae data are made available to support future studies.

## ﻿Introduction

Morphological data on plant species are crucial for scientific research in taxonomy, specimen identification, development of identification tools, and ecological and evolutionary studies that link morphology to occurrence, resistance, invasiveness, and rates of diversification and evolution.

Whereas DNA sequence data are routinely published in central repositories such as Genbank (https://www.ncbi.nlm.nih.gov/genbank/), and metadata of biological collection specimens are aggregated in portals such as the Global Biodiversity Information Facility (https://www.gbif.org/) and the Atlas of Living Australia (https://ala.org.au/), no comparable resource exists for morphological data. In Australia, AusTraits is a database that collects ecological and morphological trait data for plants (Falster et al. 2021). However, without the formal expectation that researchers deposit such data from their studies in a publicly accessible way, the coverage of such databases currently remains patchy. Morphological data remain scattered across individual species descriptions and taxonomic revisions published in journals and flora treatments. A first step in studies of taxonomic groups that require a comprehensive data table of characters for individual study species is therefore often the extraction of such data from the literature.

A variety of approaches have been explored to extract morphological data from taxonomic descriptions more efficiently than through manual transcription. They include Natural Language Processing (Endara et al. 2018) and “lower-tech”, scripted processing (Coleman et al. 2023). Due to the many technical terms, synonyms for the same character or character state, the potential for error or simplification in descriptions, and the complexity of natural organisms, these methods generally require the use of exhaustive glossaries of botanical terms and their synonyms to perform well.

Large Language Models (LLMs), which have come to broad public attention since 2023 (Zhao et al. 2024), constitute another promising tool for the extraction of data from text (Dagdelen et al. 2024). Trained on large amounts of text, they are extremely versatile and can be used for tasks ranging from spotting errors (Al-Garaady and Mahyoob 2023) to assisted coding (Liu et al. 2024). A recent study explored the use of an LLM as part of a larger Natural Language Processing pipeline and reported an error rate of 10.3% (Domazetoski et al. 2025) but was limited to five life history traits found in online databases. Another used the LLM Mistral as part of a pipeline to convert text descriptions from databases into structured data libraries and reported precision and recall scores of between 70% and 81% for three datasets (Marcos et al. 2025). However, the authors noted that a major limitation remains the limited availability of trait databases like those they used in their study.

LLMs must be used with care, as they do not understand concepts as humans do (Mitchell and Krakauer 2023) and are indifferent to the truth of their outputs (Hicks et al. 2024). In addition, their use in writing is morally questionable, as they were trained on partly unlicensed material and often plagiarise aspects of the training data or may even reveal sensitive information (Lee et al. 2023). However, LLMs have been shown to work well for text mining, where they are not being asked to provide information from their potentially misunderstood or unlicensed training data but merely reformat part of the query (Wan et al. 2024).

Here, we tested the feasibility of using Optical Character Recognition and an LLM to efficiently extract morphological characters from species descriptions and revisions in taxonomic journals, and from flora treatments of varying age and level of detail. As the study group, we used Australian Asteraceae (daisy or aster family), the area of expertise of the first author. We present the resulting data table as a resource for studies in Asteraceae and discuss cha­llenges of the process and how it could be adapted to other taxonomic groups.

## ﻿Materials and methods

### ﻿Sampling and preparation

Traits were extracted from taxonomic treatments of genera of Australian Asteraceae, including both journal papers and flora contributions (Burbidge 1958, 1982; Lander and Barry 1980; Dunlop 1981; Jessop 1981; Short 1983, 1985, 1986, 1989, 1990a, 1990b, 1995, 2000a, 2000b, 2014, 2015; Marchant et al. 1987; Wilson 1987, 1989b, 1989a, 1992d, 1992b, 1992c, 1992a, 2002; Short and Wilson 1990; Everett and Doust 1992a, 1992b, Everett and Thompson 1992; Holzapfel 1994; Forbes and Morris 1996; Hunger 1996, 1997; Gray and Given 1999; Lepschi 1999; Rozefelds 2002; Leeson and Rozefelds 2003; Keighery 2004; Thompson 2004c, 2004a, 2004b, 2005a, 2005b, 2006; Holland and Funk 2006; Wilson and Wilson 2006; Walsh 2007, 2014; Bean 2011, 2020; Messina et al. 2013, 2014; Jeanes 2015, 2020; Lyons and Keighery 2015; Collins et al. 2022). Most of the papers had been digitised and were downloaded from journal websites or the Biodiversity Heritage Library (https://www.biodiversitylibrary.org). Some were scanned manually from paper versions and converted into PDFs by the first author.

**Figure 1.**
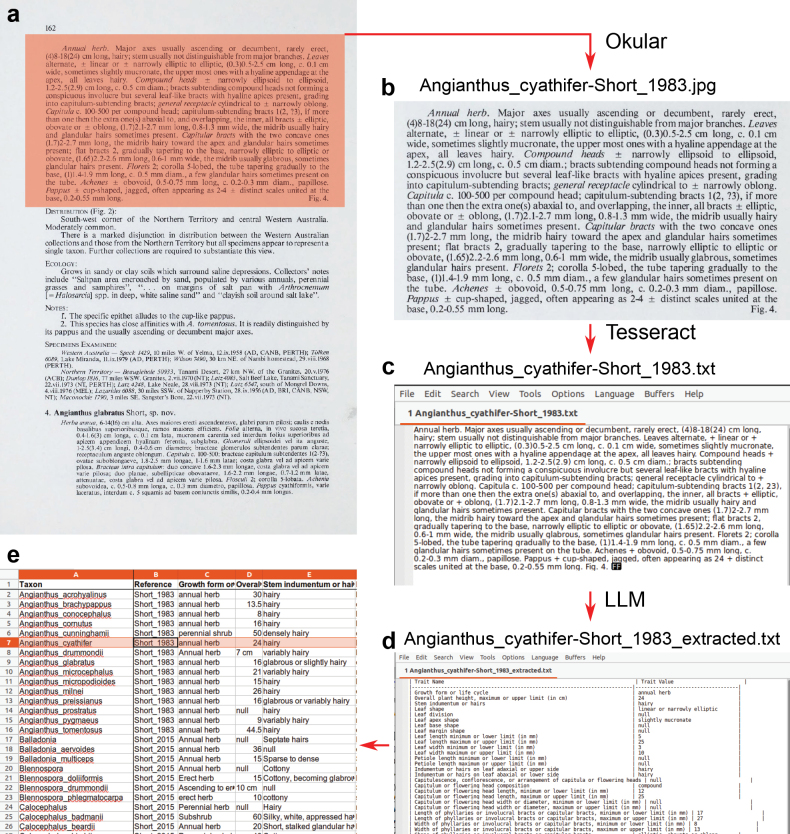
Workflow for mining of morphological data from taxonomic publications, using Short (1983) as an example. a. Description in the context of the page; b. Description copied into image file; c. Text of description extracted from image file using Optical Character Recognition; d. Output of Large Language Model (LLM) presenting character data from the description as a table; e. Detail of table collecting data from multiple descriptions, with column headers added manually. On Windows computers, Adobe Acrobat was used instead of Okular.

Many PDFs of papers available online either contain text or have text superimposed on the scanned image that was produced by Optical Character Recognition (OCR) during or after scanning. However, these results were of variable quality, making it impossible to rely on embedded text in the PDFs. Due to this, and to make the procedure consistent between both pre-existing PDFs and scanned documents, the character extraction process started in nearly all cases from images (Fig. 1).

On a Linux computer, taxonomic descriptions were cropped from the documents using the Area Selection: Image: Copy to Clipboard function of Okular v1.9.3 (https://okular.kde.org). Images were pasted into the GNU Image Manipulation Program v2.10.30 (GIMP, https://www.gimp.org) and then assembled into one image per description if they had run across more than one page. Images were exported in JPG format. On Windows computers, taxonomic descriptions were turned into images by taking screen shots of the documents opened in Adobe Acrobat v. 2024.005.20399. They were pasted into Paint for Windows 10 v. 22H2 and assembled into one image per description if they ran across more than one page. In both cases, finished images were exported in JPG format under the file naming convention “Genus_species-Author_year.jpg” to connect the taxon and document to the image for later processing. Several descriptions were stored in the same folder and batch-processed in subsequent steps.

### ﻿OCR and LLM

OCR was conducted using Tesseract v4.1.1 (Smith 2007). The resulting text for each image was automatically cleaned of line breaks and double spaces, and em-dashes were converted into dashes to avoid character coding issues. Cleaned descriptions were saved as plain text files under the same naming convention as the images.

An unpublished taxonomic treatment of *Rhodanthe* DC. by Paul Wilson and intended for the Flora of Australia was kindly provided by the Australian Biological Resources Study. It is the only document where no JPGs were created, and the process instead started from plain text files.

Characters were extracted from each description text using API calls to ChatGPT 4o (Omni). The prompt was constructed by concatenating (1) the taxonomic description, (2) an instruction text, and (3) the list of characters. The final instruction text used after the conclusion of exploratory queries was:

“Extract traits from the above text and organise them into a table with the trait name in the first column and the trait value in the second column. Follow the order of traits exactly as provided; do not skip any trait but use ‘null’ for missing information instead; and do not add any characters not specified in the list of traits. The list of traits is as follows:”

The specifications became necessary after exploratory calls to the LLM had shown that it would very rarely transpose the table or add a character that had not been requested, most commonly the chromosome number of a species. The list of characters included alternative names for characters, e.g., capitular bracts and paleae, potential character states for many characters, and the requested unit for numerical characters (Table 1). Length/width characters were broken up into separate entries for minima and maxima.

**Table 1.** List of characters extracted from taxonomic descriptions. Character group, character number, and bolding were not part of the prompt and are provided only to make the table more legible to the reader.

Character group	No.	Verbatim character description in prompt
Growth, stem, capitulescene	1	**Growth form or life cycle** (e.g., annual herb, perennial herb, subshrub, shrub, tree);
	2	**Overall plant height**, maximum or upper limit (in cm);
	3	**Stem indumentum** or hairs (e.g., glandular, eglandular, woolly, cottony, arachnose, lanate, septate hairs, or glabrous);
Leaf	4	**Leaf shape** (e.g., linear, lanceolate, ovate, elliptic, obovate, oblanceolate);
	5	**Leaf division** (e.g., lobed, compound, palmate, pinnate, bipinnate);
	6	**Leaf apex** shape (e.g., acute, obtuse, rounded, acuminate, attenuate);
	7	**Leaf base** shape (e.g., acute, obtuse, rounded, attenuate, cordate);
	8	**Leaf margin** shape (e.g., serrate, crenate, undulate, entire);
	9	**Leaf length minimum** or lower limit (in mm);
	10	**Leaf length maximum** or upper limit (in mm);
	11	**Leaf width minimum** or lower limit (in mm);
	12	**Leaf width maximum** or upper limit (in mm);
	13	**Petiole length minimum** or lower limit (in mm);
	14	**Petiole length maximum** or upper limit (in mm);
	15	**Indumentum** or hairs on **leaf adaxial** or upper side (e.g., glandular, eglandular, woolly, cottony, arachnose, lanate, septate hairs, or glabrous);
	16	**Indumentum** or hairs on **leaf abaxial** or lower side (e.g., glandular, eglandular, woolly, cottony, arachnose, lanate, septate hairs, or glabrous);
Growth, stem, capitulescene	17	**Capitulescence**, conflorescence, or arrangement of capitula or flowering heads (e.g., paniculate, corymbose, racemose, solitary capitula);
Capitulum and bracts	18	Capitulum or flowering head composition (**simple versus compound** [also known as glomerule]);
	19	Capitulum or flowering head **length, minimum** or lower limit (in mm);
	20	Capitulum or flowering head **length, maximum** or upper limit (in mm);
	21	Capitulum or flowering head **width or diameter, minimum** or lower limit (in mm);
	22	Capitulum or flowering head **width or diameter, maximum** or upper limit (in mm);
	23	**Length of phyllaries** or involucral bracts or capitular bracts, **minimum** or lower limit (in mm);
	24	**Length of phyllaries** or involucral bracts or capitular bracts, **maximum** or upper limit (in mm);
	25	**Width of phyllaries** or involucral bracts or capitular bracts, **minimum** or lower limit (in mm);
	26	**Width of phyllaries** or involucral bracts or capitular bracts, **maximum** or upper limit (in mm);
	27	**Shape of phyllaries** or involucral bracts or capitular bracts;
	28	**Margin of phyllaries** or involucral bracts or capitular bracts;
	29	**Colour of phyllaries** or involucral bracts or capitular bracts;
	30	**Indumentum** or hairs **on phyllaries** or involucral bracts (e.g., glandular, eglandular, woolly, cottony, arachnose, lanate, septate hairs, or glabrous);
	31	Receptacle bracts or **paleae present** (yes or no);
Floret	32	**Ray florets** or ligulate florets **present** (yes or no);
	33	Female-only **filiform florets present** (yes or no);
	34	Bisexual, **tubular disk florets present** (yes or no);
	35	**Colour of rays**, ray florets, or ligules;
	36	**Colour of disk florets**;
Cypsela	37	**Cypsela** or achene **length**, **minimum** or lower limit (in mm);
	38	**Cypsela** or achene **length**, **maximum** or upper limit (in mm);
	39	**Cypsela** or achene **width** or diameter, **minimum** or lower limit (in mm);
	40	**Cypsela** or achene **width** or diameter, **maximum** or upper limit (in mm);
	41	**Cypsela** or achene **shape** (e.g., cylindrical, ovoid, obovoid, fusiform);
	42	**Cypsela** or achene **flattened** or compressed (yes or no);
	43	**Cypsela** or achene **winged** (yes or no);
	44	**Cypsela** or achene has longitudinal ribs (yes or no);
	45	**Cypsela** or achene surface **colour**;
	46	**Cypsela** or achene hairs or **indumentum** (e.g., papillose, twin hairs, myxogenic hairs, glandular, or glabrous);
Pappus	47	**Pappus present** (yes or no);
	48	**Pappus type** (bristles, scales, awns, crown);
	49	**Pappus ornamentation** (barbellate, plumose, smooth);
	50	**Pappus length, minimum** or lower limit (in mm);
	51	**Pappus length, maximum** or upper limit (in mm).

### ﻿Processing of LLM responses

Responses obtained for each taxonomic description were saved as plain text files with “_extracted” added to the file name. The script automating the process parsed the response text by searching for lines starting with |, indicating they are part of the table the LLM had been asked to produce, and skipping the first two, which represented the header and a horizontal line separating table header and body. It then split all remaining table lines on “| “ and searched for entries in the second column, assuming that characters are returned in the same order as provided in the prompt.

Character data were appended to a table collecting data from all descriptions processed in the same batch run, with the first half of the original image file name as the first column (taxon) and the second half of the file name as the second column (source document). This table was then saved as a text file in tab separated value format (TSV), serving as the final output of each batch process. Several such tables were manually combined into the master table presented with this paper (DOI: 10.25919/8gd0-5v96).

Custom-written Python 3 scripts used in this study are deposited at https://bitbucket.csiro.au/projects/NRCA/repos/image-classification/browse/trait_mining, with empty placeholders for the user’s subscription key and API endpoint.

### ﻿Processing and cleaning of results

Many of the sources of morphological descriptions used outdated taxonomy. Names were updated using the vlookup function in LibreOffice v7.3.7.2 Calc based on an extract of the Australian Plant Census (https://biodiversity.org.au/nsl/services/search/taxonomy, accessed 5 August 2021) for Asteraceae and supplemented with more recently published names. The accepted name column was left blank for some taxa that have been more narrowly circumscribed or split since a source was published, e.g., in the cases of genus-level morphological descriptions of *Gnaphalium* L., *Helichrysum* Mill., and *Helipterum* DC.

Character data were harmonised using OpenRefine v3.5.2 (Ham 2013) by creating facets with the key collision method and the fingerprint keying function to harmonise character states (e.g., “acute to obtuse”, “acute or obtuse”, and “obtuse or acute”). Character states were further mass-edited to make all text lower-case, to remove the ~ (approximate) symbol, to remove units from numeric characters, to correct ± that had been misread as +, and to correct “No data” to null.

### ﻿Accuracy

To test the accuracy of results, 109 descriptions were randomly chosen and their original text compared against the table populated by processed ChatGPT 4o outputs. Errors were scored into the following categories: missing (information was not retrieved that was present in the description), partial (not incorrect, but only some of the available information relevant to the trait was retrieved), hallucinated (information was ‘guessed’ or invented that was not present in the description), wrong (straightforward false information despite the correct information being present in the description), wrong but understandable given confusing wording of the description (e.g., the term “compound” in capitulescence structures ‘tricking’ the LLM into concluding that the capitulum is compound), misapplied (e.g., calycular bract dimensions or capitulum dimensions retrieved for involucral bract dimensions), unit error (e.g., cm where mm were specified), and OCR errors.

Error rates were also evaluated separately for character groups (Table 1) to explore how the LLM performed on different organ types: growth / stem / capitulescence, leaf, capitulum, floret, cypsela, and pappus. They were further evaluated separately for character classes to explore performance on descriptive, numeric, and binary traits: dimensions (numeric, with the expected unit specified in the prompt), the presence of an organ (with yes or no specified in the prompt as the expected reply), shapes in the widest sense (with potential options sometimes specified in the prompt), indumentum (with potential options always specified in the prompt), and colour (no suggestions specified in the prompt).

### ﻿Reproducibility

To test the reproducibility and consistency of results, the same 109 descriptions as used for accuracy testing were processed again with an LLM using the same prompts as before. First, processing was repeated using the same model, ChatGPT 4o. Results were compared after harmonising all missing data fields to ‘null’ by counting the number of fields where both replicates returned missing data, where one replicate returned missing data and the other returned data, and where both replicates returned exactly the same data. Fields for which both replicates returned data with different text were compared visually to determine if the difference is substantive but at least the correct type of character (e.g., 10 mm *versus* 40 mm), due to a misunderstanding of the query in one of the two replicates (e.g., retrieving capitulum shape instead of the requested phyllary shape), due to different levels of precision, variation, or detail being retrieved (e.g., indumentum “woolly, glandular” *versus* only “woolly”), or not substantive (e.g., capitulescence “corymbose clusters” *versus* “corymbose”).

### ﻿Open source models

In a second test, the same 109 descriptions were processed with an open source model run locally in Ollama v0.9.6 (https://ollama.com). We explored the behaviour of Mistral v0.3, Deepseek-R1-0528 (Bi et al. 2024), and OpenChat v3.5-0106 (Wang et al. 2024) with a single inference and selected OpenChat for the full replicate test. Evaluation was conducted as for the replicate of ChatGPT 4o, but we scored additional categories of mismatch between the replicates as follows: OpenChat retrieved large amounts of information irrelevant to the requested trait, OpenChat united minimum and maximum dimensions into one table row, OpenChat provided numerical value in the wrong unit (e.g., cm instead of the requested mm), and OpenChat skipped a trait. The first of these unavoidably involved some subjective judgement. We did not score for this category if we judged that in the given context, the presence of the additional information was understandable as only a minor misunderstanding of the query as would be experienced by a non-expert human, or if the additional information was limited and would likely be easy to remove during subsequent processing of the data. We scored for this category if the additional information was clearly irrelevant to the trait and belonged elsewhere in the table (e.g., cypsela indumentum “densely or sparsely sericeous; ribs 10-13”, as presence of ribs was queried separately) or if it was excessive (e.g., lengthy description of an organ in response to the query regarding its presence with suggested options of “yes” and “no”).

## ﻿Results

In total, we extracted data from 1,121 profiles. Following currently accepted taxonomy and after accounting for taxa with descriptions in multiple sources, they comprise data for 95 genera and 838 species or infraspecific taxa, totalling 945 taxa. 50.5% of the originally extracted profile × character matrix constituted missing data. After cleaning and harmonising character states, 51.1% constituted missing data. However, end-users of the data would be able to populate some missing data cells in species descriptions with the character state provided in the genus description from the same source.

OCR with Tesseract performed mostly reliably, even for documents that had been scanned slightly at an angle, showed some curvature of the text towards the spine of the volume, or were low resolution (e.g., lowercase letters only nine pixels tall). However, it struggled with fractions presented as a single character (e.g., ½), frequently reading them as percentage signs, and with ±, which was consistently misread as +. In rare cases, Tesseract overlooked a decimal point in numerical characters or a dash/hyphen in minimum–maximum ranges or made other errors as specified below.

The LLMs were asked to return data in a tabular format and nearly always returned text in standard markdown format by using columns of text separated by pipes (|), and a header row separated from its corresponding body rows by a line of hyphens (-). In a single case, ChatGPT 4o returned the table in HTML format, and in another case, in LaTeX. Both were returned in the usual markdown format when the queries were repeated verbatim. In a single case, ChatGPT skipped one character in the table instead of scoring it as null. In addition, the responses of ChatGPT showed a variety of more common inconsistencies:

Character names were either presented as in the query or shortened. If not shortened, long character names sometimes caused line breaks in the first column, so that one table row would run over two text lines in the output. In most cases, however, each table row equated to one line of text. Sometimes, additional text would be included above or below the table, e.g., “Note: Traits for which no information was provided directly in the description are marked as null.”

Text characters were retrieved rather inconsistently even where the prompt had provided examples of possible character states. For example, despite the prompt specifying “Pappus ornamentation (barbellate, plumose, smooth)”, the response for *Gnephosisnewbeyi* P.S.Short read “Entire bristle-like elements”.

Numerical characters either did or did not include the unit, and text character states, including “null”, were arbitrarily either capitalised or not. Very rarely, null was presented in all capital letters, and in one case, it was replaced with “No data”.

### ﻿Accuracy

Comparison of 109 descriptions against ChatGPT results revealed 200 errors for a total of 5,559 cells (Table 2). Of the 2,638 cells not containing data, 32 (1.2%) should have been populated with data that were present in the description. Of the 2,921 cells containing data, 107 (3.7%) contained misapplied data, 25 (0.9%) wrong data, 18 (0.6%) partial or imprecise data, 7 (0.2%) hallucinated data, 5 (0.2%) mistakes that were likely caused by confusing descriptions, 4 (0.1%) unit errors, and 2 (0.1%) OCR errors that the LLM had carried over (Table 2), for a total error rate of 5.8% across cells containing data.

In terms of relative frequency of errors, of the 200 observed errors, 53.5% were misapplication of data to the wrong trait, 16.0% were failure to retrieve information that was present, 12.5% were false data, 9.0% were partial or imprecise data, 3.5% were hallucinations, 2.5% were caused by confusing wording in the source text, 2.0% were unit errors, and 1.0% were OCR errors.

**Figure 2.**
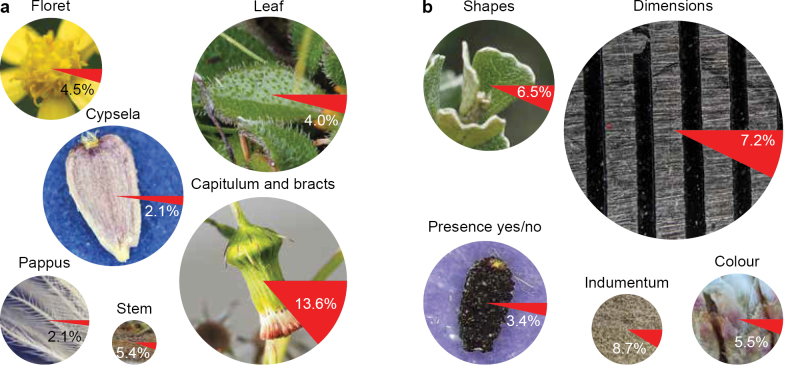
Visualisation of error rates for different a. character groups and b. character classes as inferred from data extracted for 109 descriptions. Circle sizes are proportional to the number of cells containing data, and the red slice indicates error rate. Error rates are particularly high for capitulum and bract characters, because the LLM did not ‘understand’ the distinctions between the involucre as a whole, individual involucral bracts (phyllaries), and the calycular bracts of tribe Senecioneae. Error rates are low for cypsela and pappus characters and for questions regarding the presence or absence of organs (e.g., ray florets, receptacle bracts, pappus).

Analysis of error rates by character group showed that most errors – specifically, misapplication of data – affected capitulum and bract characters. This led to a much higher error rate in that character group (13.6%) compared to the other five groups, which had error rates between 2.1% and 5.4% (Table 2, Fig. 2). The underlying errors were mostly confusion between individual involucral bracts and the involucre as a whole and, in tribe Senecioneae, confusion between calycular bracts and involucral bracts (phyllaries).

For character classes, error rates were highest for indumentum (8.7%), despite the inclusion of potential character states in the prompt, followed by dimensions (7.2%), shapes (6.5%), colour (5.5%), and binary presence/absence (3.4%) (Table 2, Fig. 2). The error profile for indumentum stood out by the relatively large percentage of partial or imprecise data (3.1% *versus* 0.0–1.8% in other classes), i.e., the LLM frequently did not retrieve all types of trichomes mentioned in the source text.

**Table 2.** Evaluation of errors made by Optical Character Recognition and text parsing by a Large Language Model for different character groups and classes. The same traits or characters are split into organ types and then again into character classes. Error rates are calculated relative to the number of cells with data except in the case of ‘missing’, where they are calculated relative to cells not containing data, i.e., the fraction of cells without data that are wrong, because data for them should have been retrieved from the text. Note the exceptionally high rate of data being misapplied to the wrong cell for capitulum and bract characters, as the LLM confused different kinds of bracts and retrieved capitulum length as phyllary length or *vice versa*. Error rates are intermediate for growth, stem, and capitulescence, leaf, and floret characters and lowest for cypsela and pappus characters. Also of note is that indumentum characters are more affected than other kinds of characters by partial or imprecise information extraction, e.g., retrieval of only some of several types of trichomes mentioned in the source text.

Character groups / classes	Growth, stem, capitulescence	Leaf	Capitulum and bracts	Floret	Cypsela	Pappus	Shapes	Dimensions (numeric)	Indumentum	Colour	Presence (yes/no)
**Cells with data**	74	675	777	310	559	243	491	1292	161	253	441
**Misapplied to wrong trait**	2 (2.7%)	14 (2.1%)	83 (10.7%)	2 (0.6%)	2 (0.4%)	4 (1.6%)	21 (4.3%)	70 (5.4%)	7 (4.3%)	9 (3.6%)	0 (0.0%)
**Hallucinated**	0 (0.0%)	0 (0.0%)	0 (0.0%)	3 (1.0%)	4 (0.7%)	0 (0.0%)	0 (0.0%)	0 (0.0%)	0 (0.0%)	3 (1.2%)	4 (0.9%)
**False**	1 (1.4%)	0 (0.0%)	16 (2.1%)	7 (2.3%)	0 (0.0%)	1 (0.4%)	2 (0.4%)	11 (0.9%)	2 (1.2%)	1 (0.4%)	9 (2.0%)
**Partial or imprecise**	1 (1.4%)	9 (1.3%)	4 (0.5%)	0 (0.0%)	4 (0.7%)	0 (0.0%)	9 (1.8%)	3 (0.2%)	5 (3.1%)	1 (0.4%)	0 (0.0%)
**Wrong due to confusing source text**	0 (0.0%)	0 (0.0%)	3 (0.4%)	2 (0.6%)	0 (0.0%)	0 (0.0%)	0 (0.0%)	3 (0.2%)	0 (0.0%)	0 (0.0%)	2 (0.5%)
**Unit error**	0 (0.0%)	4 (0.6%)	0 (0.0%)	0 (0.0%)	0 (0.0%)	0 (0.0%)	0 (0.0%)	4 (0.3%)	0 (0.0%)	0 (0.0%)	0 (0.0%)
**OCR error**	0 (0.0%)	0 (0.0%)	0 (0.0%)	0 (0.0%)	2 (0.4%)	0 (0.0%)	0 (0.0%)	2 (0.2%)	0 (0.0%)	0 (0.0%)	0 (0.0%)
**Total error rate on data**	4 (5.4%)	27 (4.0%)	106 (13.6%)	14 (4.5%)	12 (2.1%)	5 (2.1%)	32 (6.5%)	93 (7.2%)	14 (8.7%)	14 (5.5%)	15 (3.4%)
**Cells without data**	362	742	749	235	531	302	817	1106	384	183	431
**Missing (data not retrieved despite present in text)**	2 (0.6%)	4 (0.5%)	7 (0.9%)	6 (2.6%)	7 (1.3%)	6 (2.0%)	5 (0.6%)	15 (1.4%)	2 (0.5%)	1 (0.5%)	9 (2.1%)

### ﻿Reproducibility

**Figure 3.**
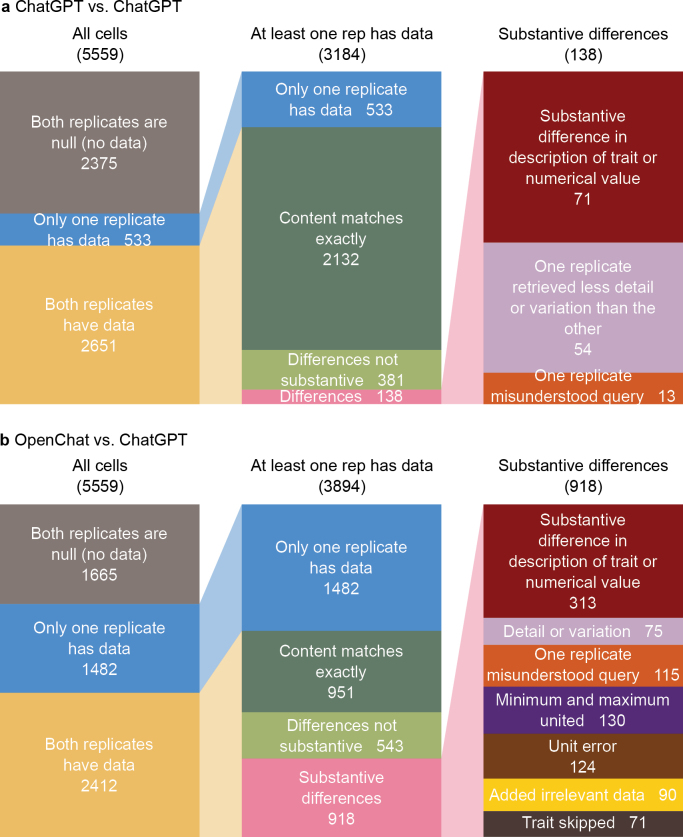
Visualisation of reproducibility of data extraction from taxonomic descriptions using a. the same LLM as before, ChatGPT 4o, and b. an open source model run locally, OpenChat. OpenChat misunderstood more trait queries, frequently united minima and maxima into one table row, and sometimes provided large amounts of information irrelevant to a trait, or skipped traits entirely.

Queries for the same 109 descriptions were repeated exactly as before with ChatGPT 4o. Results are summarised in Suppl. material 1. Of the 5,559 data cells subjected to pair-wise comparison, 2,375 (42.7%) were empty in both replicates. However, only 2,651 (47.7%) cells contained data in both replicates, whereas 533 (9.6% of total cells, or 16.7% of the cells containing data for at least one replicate) contained data in one replicate but not the other (Fig. 3a).

Of the cells containing data in at least one replicate, the content matched exactly in 2,132 (67.0%) cases, and another 381 (12.0%) cells showed no meaningful difference after visual evaluation, for a total of 78.9% reproducibility for cells containing data in at least one replicate. Minor sources of discrepancies were substantive differences in character description or numerical values (71 cells, 1.3%), one replicate providing less detail or precision than the other (54 cells, 1.7%), and one replicate misunderstanding what information is being requested (13 cells, 0.4%) (Fig. 3a).

### ﻿Open source models

When tested on one description, DeepSeek-R1 inexplicably retrieved most numerical values in µm (e.g., “500” instead of the “0.5 mm” found in the source text) despite mm being specified in the prompt. Mistral ignored direct instructions not to unite minimum and maximum dimensions of the same organ into one table row, even when we tried alternative prompts, including an empty example table and the instruction to use it as a template. OpenChat produced the best results for the single query and was therefore chosen for full replicate test. Results are summarised in Suppl. material 1. Despite the promising first test, OpenChat unpredictably skipped traits for 40 of the 109 descriptions, often, like Mistral, united minimum and maximum values for the same organ into one row, and produced formatting glitches in many of its markdown tables. Instead of consistently using “null” for missing data as instructed, OpenChat frequently used alternatives such as “Not specified”, “Not applicable”, “N/A”, “-”, “Not provided”, or “Not mentioned”. Because of this, its responses would be functionally useless for scripted processing of taxonomic descriptions at scale and required lengthy manual editing to enable the comparison of consistency with results obtained from ChatGPT. All data for one description (*Podothecawilsonii* P.S.Short) were scored as wrong because the table produced by OpenChat was too incoherent to be worth correcting manually.

Of the 5,559 data cells subjected to pair-wise comparison between ChatGPT and OpenChat, 1,665 (30.0%) were empty in both replicates, 2,412 (43.4%) either contained data in both replicates or were skipped by OpenChat (as opposed to being marked as null or some synonym), and 1,482 (26.7% of total cells, or 38.1% of the cells containing data for at least one replicate) contained data in one replicate but not the other (Fig. 3b).

Of the cells containing data in at least one replicate, the content matched exactly in 951 (24.4%) cases, and another 543 (13.9%) cells showed no meaningful difference after visual evaluation, for a total of 38.4% reproducibility of ChatGPT results with OpenChat for cells containing data in at least one replicate (Fig. 3b). Apart from one model returning data where the other did not (see previous paragraph), the following sources of discrepancies were observed in order of descending frequency: meaningful differences in character description or numerical values (313 cells, 8.0%), OpenChat uniting minimum and maximum values into one table row (130 cells, 3.3%), OpenChat providing a numerical value in a different unit than requested (124 cells, 3.2%), one replicate misunderstanding what information is being requested (115 cells, 3.0%), OpenChat providing large amounts of information irrelevant to the requested trait (90 cells, 2.3%), one replicate providing less detail or precision than the other (75 cells, 1.9%), and OpenChat entirely skipping a trait (71 cells, 1.8%).

It should be noted that this comparison regards reproducibililty of ChatGPT results with an open source model and, outside of where OpenChat has been explicitly mentioned, not necessarily which of the two models provided accurate results. This means that even in cases where both models provided the same response, that response may sometimes be false. Conversely, in many of the cases where there was a discrepancy in the amount of detail or variation provided by both models, OpenChat provided the larger amount.

## ﻿Discussion

The combination of OCR and data extraction using an LLM allowed us to process morphological data from hundreds of taxonomic descriptions in a fraction of the time (a few days) compared to manual methods. Most of the initial human effort was spent on cropping out descriptions and refining the scripts to better handle unexpected LLM outputs. Subsequently, several hours were required to clean and harmonise character state names across the resulting data table and remove data that were incorrectly assigned to columns. However, while we consider our approach promising and at least easily transferable to other taxonomic groups by replacing the list of characters we used, the unpredictable nature of LLM outputs makes it difficult to use without further refinement and careful checking to ensure accuracy.

Inconsistencies in the LLM outputs made parsing them difficult and required iterative adaptation of the script, e.g., to skip empty lines in the second column caused by line breaks in the first column. A potential solution for this particular problem would be to request the LLM to output data in JSON format. However, the inconsistent naming of characters in the markdown-formatted responses suggests that the same could have happened in JSON fields. Therefore, it seemed easier to base processing of the responses on the order of characters in the table, which exploratory queries had demonstrated to be more consistent than formatting.

These and other inconsistencies in the text-mining outputs, most notably the cases where outputs were unexpectedly provided in HTML and LaTeX formats, made it necessary to repeat LLM queries for individual profiles. The tendency of LLMs to return responses formatted in unexpected ways makes it hard to integrate them into reliably automated workflows, as it is impossible to predict all potential such problems in advance. This means there is a trade-off between the ability of an LLM to extract information from a great variety of texts and the predictability but lower flexibility of script-based approaches (Coleman et al. 2023).

These issues could be addressed by further refining the prompts, for example, by asking the LLM to explicitly provide the output in a markdown tabular format. However, for consistency after such refinements, all data should ideally be re-processed with no guarantee that any given refinement would result in 100% consistent outputs, as demonstrated by ChatGPT in one case, and OpenChat in numerous cases, ignoring the explicit instruction not to skip any characters. Each iteration of refining prompts therefore diminishes the time savings potentially made by using this type of workflow. That being said, future, larger studies applying this approach can build on the observations made in this study already in their design phases.

Although use of OCR and LLM largely automated the population of a character-by-taxon table, a time-consuming step in our approach remains the cropping of morphological descriptions from the publications. Extracting information directly from PDF files of the publications could potentially remove the need for this manual step (Ma et al. 2024). However, we did not explore this option here, mainly because, as discussed earlier, where PDFs of older taxonomic publications contained text generated by OCR during digitisation, it was sometimes garbled. Errors in assigning text to the correct columns and rows are known to mislead LLMs (Gerard 2025).

Most of the errors made by the LLMs in assigning information to the correct character resulted from apparent confusion between different kinds of bracts and between involucral bracts and the involucre as a whole. We did not explore potential solutions more deeply, but it is possible that the LLMs’ “understanding” of Asteraceae morphology is too shallow to ensure accuracy in these cases, as suggested by the more reliable results for less taxon-specific characters such as leaf length and indumentum.

This study was designed to test the utility and feasibility of extracting morphological characters with OCR and LLMs in their current readily available states with minimum input and modifications, and to identify any common pain points which would need to be addressed when undertaking a larger project of this nature. We identified several consistent OCR issues (e.g., ± and fractions) that must be considered. The most challenging LLM issue appears difficult to solve without more in-depth approaches such as modifying the underlying model or applying other advanced techniques.

The core of this issue is the LLM’s “understanding” of key concepts and terminology. There are several approaches currently available which can address this. For example, Low Rank Adaptation (LORA) (Hu et al. 2021) and Parameter-Efficient Fine-Tuning (PEFT) methods (Zhang et al. 2024) are both techniques for adapting an LLM in a cost-effective way. Alternatively, Retrieval-Augmented Generation (RAG) (Gao et al. 2024) provides a way of supplying additional specific data for the LLM to search through prior to responding to the query prompt. In the case of RAG, additional decisions/expertise are required to integrate the source data and architectures used, but this is a clear pathway to improving results by providing a more detailed understanding of botanical concepts to the LLM before requesting the extraction of data from a description. These approaches all have the potential to significantly improve results but also come with additional costs in time, compute, and other resources to develop the workflow and require additional training or reference data sources.

We previously conducted exploratory RAG of information such as the geographic occurrence of species from various scientific publications on Asteraceae tribe Gnaphalieae and found that responses were wrong to two of our four queries despite the relevant information being available in the input publications (unpubl. data). It should be noted, however, that we had used a more compact model with more limited computational resources than ChatGPT 4o in our exploratory RAG (Candel et al. 2023; Tunstall et al. 2023), so that better results may be achievable, at least with readable PDF files. Conversely, the poorer performance of an open access model running on a local computer compared to ChatGPT 4o in this study suggests that researchers may have to invest more time into manual data cleaning when using such models under restricted access to high-performance computing. As memory and GPU technology advance, hardware limitations that currently restrict the performance of open source models on local machines may diminish. This could allow researchers to run larger, more sophisticated models with greater accuracy and efficiency, making local open source solutions increasingly viable compared to commercial alternatives.

The character data extracted here from 1,121 taxonomic descriptions of Australian Asteraceae are available on the CSIRO Data Access Portal at DOI: 10.25919/8gd0-5v96. As they comprise information on life cycle or growth form, capitulum composition, absence or presence of paleae and pappus, and pappus ornamentation, they will be useful in taxonomic studies on the circumscription of genera and to understand the impact of character change in evolutionary history, e.g., by tracing character states on phylogenetic trees. However, capitulum and bract characters should be used with caution, given the relatively high error rate compared to the other character groups, and especially in tribe Senecioneae due to potential of confusion between calycular and involucral bracts.

In cleaning the data before publication, we aimed to strike a balance between harmonising the character states across descriptions and leaving the original data intact. For complex characters such as indumentum and capitulescence structure, it is difficult to predict what any end-user of the data would be interested in, so the available information has therefore been left to vary greatly (e.g., “arachnose” in one case, “dense minute indumentum of unicellular uniseriate or biseriate conical hairs” in another). We also did not correct instances of taxonomists wrongly describing corymbose panicles as “cymes”, as this conceptual error is widespread in the community, and the correction may be confusing to some end-users. Taken together, this means that end-users of the collated data table need to invest additional effort to convert complex characters into simple categories for quantitative analysis.

## ﻿Additional information

### Use of AI

No AI was used to write the manuscript.

### Author ORCIDs

Alexander N. Schmidt-Lebuhn https://orcid.org/0000-0002-7402-8941

Nunzio Knerr https://orcid.org/0000-0002-0562-9479

### Data availability

The table of morphological data for Australian Asteraceae and results of accuracy and reproducibility tests are available on the CSIRO Data Access Portal at DOI: 10.25919/8gd0-5v96. Custom-written Python 3 scripts used in this study are deposited at https://bitbucket.csiro.au/projects/NRCA/repos/image-classification/browse/trait_mining, with empty placeholders for the user’s subscription key and API endpoint.

## Supplementary materials

Supplementary material 1 Morphological data matrix of 838 Australian Asteraceae text-mined from taxonomic descriptions Data type xlsx Explanation note Spreadsheet file containing 51 columns of morphological data for 95 genera and 838 species or infraspecific taxa of Australian native and introduced Asteraceae. The first tab contains data as extracted from sources using Optical Character Recognition and a Large Language Model. The second tab contains the same data after cleaning for consistency using OpenRefine. For each taxon, a link or reference to the source is provided. https://binary.pensoft.net/file/1396813This dataset is made available under the Open Database License (http://opendatacommons.org/licenses/odbl/1.0/). The Open Database License (ODbL) is a license agreement intended to allow users to freely share, modify, and use this Dataset while maintaining this same freedom for others, provided that the original source and author(s) are credited.Alexander N. Schmidt-Lebuhn

Supplementary material 2 Results of accuracy test and reproducibility tests on 109 randomly selected descriptions Data type xlsx Explanation note A spreadsheet document comprising six tabs: (1) Results of the accuracy test, a visual comparison of 109 source descriptions against the processed results from text-mining with ChatGPT. Under the table with the morphological data, errors are categorised in a second table, and below that, errors are counted for different categories. (2) Results of repeated text-mining with ChatGPT. (3) Categorisation of mismatches between the two ChatGPT replicates, and numerical summary. (4) Results of text-mining with OpenChat. (5) Results of text-mining with OpenChat after manual editing to correct for skipped traits. (6) Categorisation of mismatches between ChatGPT and OpenChat, and numerical summary. https://binary.pensoft.net/file/1396814This dataset is made available under the Open Database License (http://opendatacommons.org/licenses/odbl/1.0/). The Open Database License (ODbL) is a license agreement intended to allow users to freely share, modify, and use this Dataset while maintaining this same freedom for others, provided that the original source and author(s) are credited.Alexander N. Schmidt-Lebuhn
